# Revisiting the Pharmacological Value of Glucagon: An Editorial for the Special Issue “The Biology and Pharmacology of Glucagon”

**DOI:** 10.3390/ijms21020383

**Published:** 2020-01-08

**Authors:** Timo D. Müller, Kirk Habegger

**Affiliations:** 1 Institute for Diabetes and Obesity, Helmholtz Diabetes Center, Helmholtz Zentrum München, German Research Center for Environmental Health (GmbH), 85764 Neuherberg, Germany; 2 German Center for Diabetes Research (DZD), 85764 Neuherberg, Germany; 3 Department of Pharmacology and Experimental Therapy, Institute of Experimental and Clinical Pharmacology and Toxicology, Eberhard Karls University Hospitals and Clinics, 72076 Tübingen, Germany; 4 Department of Medicine-Endocrinology and Diabetes and Metabolism, Comprehensive Diabetes Center, University of Alabama at Birmingham, Birmingham, AL 35899, USA

In 1921, a Canadian research team led by Frederick Banting and John Macleod succeeded in the isolation of insulin from pancreatic homogenate [1] and thereby turned juvenile-onset (type 1) diabetes from a fatal to a manageable disease. Overshadowed by the monumental importance of this discovery was the observation that their insulin preparation first notably increased blood glucose before the much-heralded drop in glycemia was observed [2]. In 1923, the same year in which Banting and Macleod were awarded the Nobel Prize in Medicine for their discovery, Charles Kimball and John Murlin identified this hyperglycemic factor and named it glucagon [3]. Secreted from the pancreatic α-cells in negative correlation to blood glucose, glucagon acts on the liver to enhance de novo glucose production and to stimulate the breakdown of glycogen [4,5,6,7,8,9,10]. Ever since, glucagon is largely recognized for its ability to oppose the hyperglycemic effects of insulin with therapeutic value largely restricted to treat severe hypoglycemia. This negative image of glucagon is further spurred by the demonstration that the inadequate postprandial suppression of glucagon secretion can contribute to hyperglycemia and the development of type 2 diabetes (T2D) [11,12,13,14,15], whereas the blocking of glucagon action improves glucose metabolism in rodents, dogs, monkeys, and humans (as reviewed in [16]). Stigmatized for being largely of diabetogenic nature, glucagon is a pleiotropic hormone with metabolic action that goes far beyond the regulation of blood glucose. 

Emerging evidence suggests that beyond its counterregulatory role in glucose homeostasis, glucagon regulates a range of actions including lipolysis, ketogenesis, fatty acid oxidation, satiety, food intake, thermogenesis, energy expenditure (EE), and bile acid metabolism. These glucagon-mediated improvements in lipid and energy metabolism may be quite desirable in patients with the metabolic syndrome. However, the exact mechanism(s) by which glucagon elicits its beneficial metabolic responses, or more importantly, how they can be balanced against glucagon-stimulated diabetogenesis, have yet to be elucidated.

However, in 2009, a novel peptide was characterized by Day et al. that combined the antidiabetic and anorexic properties of GLP-1 with the thermogenic and lipolytic effects of glucagon (GCG) in a single molecule based on the GCG backbone [17]. Intriguingly, the antidiabetic actions of GLP-1R agonism are enhanced by the catabolic and hypolipidemic properties of glucagon receptor (GCGR) agonism [17]. Importantly, this GLP-1R/GCGR mixed agonist lacked the classic acute diabetogenic effects often attributed to GCGR activity [17]. In the same month as this initial report, a similar molecule (i.e., GLP-1/GCGR single-molecule co-agonist) was reported by Pocai et al. [18]. This version was based on the oxyntomodulin sequence and contained full activity at both GLP-1 and GCG receptors and stimulated similar beneficial effects in diet-induced obese (DIO) mice as the “Day” molecule. Together, these two preclinical studies set the stage for an anti-diabetic therapeutic that included GCGR, a thought that was antithetical prior to this evidence. Following on the heels of these preclinical rodent studies, it was observed that co-administration of GCG and GLP-1 in humans stimulated energy expenditure while reducing GCGR-associated hyperglycemia [19], thus confirming the translational importance of this strategy. With the success of these early studies, it is of no surprise that another GLP-1/glucagon co-agonist, MEDI0382, displayed potent effects on weight loss, hepatic fat content, and glucose metabolism in DIO mice and cynomolgus monkeys [20]. This specific compound has since passed safety and tolerability endpoints in phase I trials [21], as well as displaying impressive metabolic benefits in phase IIa. MEDI0382 specifically reduced postprandial blood glucose, body weight, and hepatic fat content in patients with T2D [22]. More recent findings have established the value of adding the glucose-dependent insulinotropic polypeptide receptor (GIPR) agonism to that of GLP-1/GCGR [23]. Together, these findings argue for a reevaluation of glucagon as a therapeutic target, a topic highlighted in this Special Issue.

This Special Issue, “Glucagon”, contains ten review articles covering a wide range of glucagon actions, including the metabolic effects of glucagon, methodology in glucagon research, glucagon receptor signaling, and potential therapeutic avenues for glucagon (Table 1). 

Several articles in this issue discussed themes on metabolic action regulated by glucagon receptor signaling. Al-Massadi and colleagues highlight the anti-obesity effects of glucagon via its stimulation of energy expenditure and lipid metabolism while concomitantly suppressing food intake [25]. González-García et al. extend upon the topic of energy expenditure to discuss the regulation of thermogenesis following GLP-1 or GCGR agonism [26]. Of note, this review delves into potential mechanisms of brown versus beige adipose activation by these highly related hormones in the regulation of thermogenesis. Kleinert and colleagues similarly address mechanisms underlying glucagon regulation of systemic metabolism, emphasizing the role of thermogenesis in this action and highlighting important differences of glucagon’s thermogenic action depending on whether glucagon is given acutely or chronically [27]. In the final review of this topic, Zhang et al. discuss critical intracellular targets of GCGR signaling in liver [28]. This review provides convincing arguments for acetylation of energy-sensing factors in response to GCGR agonism as the intracellular mechanism(s) for glucagon-stimulated hepatic action. Similar reviews follow with an emphasis on GCGR signaling. Reviews by Al-Zamel et al. as well as Mathiesen and colleagues address regulation of glucagon production/secretion following activation of the canonical incretin axis (i.e., GIP- and GLP1-R) [29,30]. These intriguing reviews highlight the role of glucagon in an emerging class of anti-diabetic and anti-obesity therapeutics. Although described almost 100 years ago, the accurate and precise measurement of glucagon has been a constant challenge to the field. Within this issue is an important piece from Holst and Wewer Albrechtsen regarding methodologies and recommendations for measuring glucagon in circulation [31]. The two remaining reviews of this issue include detailed analyses of insulin resistance and α-cell dysfunction by Honzawa et al. [32] as well as exciting new therapeutic options for glucagon rescue of hypoglycemia via intranasal delivery by Pontiroli and Tagliabue [33].

Overall, the collection of these ten reviews provide a historical context to the field of glucagon biology. More importantly, these articles highlight the current focus of the field, and offer insight into novel directions for work on this long-known hormone.

## Figures and Tables

**Table 1:** Overview of “The Biology and Pharmacology of Glucagon”.

Authors	Title	Topics	Type
Al-Massadi et al.	Glucagon control on food intake and energy balance	Metabolic effects of glucagon	Review
González-García et al.	Glucagon, GLP-1 and thermogenesis	Metabolic effects of glucagon	Review
Kleinert et al.	Glucagon regulation of energy expenditure	Metabolic effects of glucagon	Review
Zhang et al.	Glucagon-induced acetylation of energy-sensing factors in control of hepatic metabolism	Metabolic effects of glucagon	Review
Al-Zamel et al.	A Dual GLP-1/GIP Receptor Agonist Does Not Antagonize Glucagon at Its Receptor but May Act as a Biased Agonist at the GLP-1 Receptor	Glucagon receptor signaling	Article
Janah et al. [24]	Glucagon receptor signaling and glucagon resistance	Glucagon receptor signaling	Review
Mathiesen et al.	The effects of dual GLP-1/GIP receptor agonism on glucagon secretion-a review	Glucagon receptor signaling	Review
Holst and Wewer Albrechtsen	Methods and guidelines for measurement of glucagon in plasma	Glucagon research methodology	Review
Honzawa et al.	Cell autonomous dysfunction and insulin resistance in pancreatic α cells	Glucagon production	Review
Pontiroli and Tagliabue	Therapeutic use of intranasal glucagon: Resolution of hypoglycemia	Therapeutic avenues for glucagon	Review
